# Dental Hints Department

**Published:** 1907-06

**Authors:** B. H. Teague

**Affiliations:** Aiken, S. C.


### ...DENTAL HINTS DEPARTMENT...

CONDUCTED BY B. H. TEAGUE, D. D. S., AIKEN, S. C., TO WHOM ALL CONTRIBUTIONS TO THIS DEPARTMENT SHOULD BE SENT

Keep a scrap-book of remedies etc.

Use the finest of emery flour on the natural teeth and rather coarse for pressing down plates.

Johnson & Johnson now make a sterile paper napkin for dental use. They are O. K.

Use ammonia and sapolio on the dirty bulbs of your syringe and chip blower.

Do you run with the hare and the hounds—posing before the society as transcendentally ethical and in private practice doing the meanest of dirty little things to get patients from your brother? If so quit and be a man.

Sticky Wax.—The following is a formula for sticky wax: Pure white beeswax 4 oz., pure light yellow resin 7 oz., pure gum dammar 1 oz., dye q. s. to color. Powder the resin and gum dammar and add little by little the melted beeswax.—Dental Record.

Three Useful Pointers.—To take vulcanized rubber from teeth taken off plates.—Place teeth in a small, wide-mouthed bottle containing chloroform, over night. The rubber may be removed as easily as so much charred cork.—J. W. Marshall, Cosmos.

Nausea.—To relieve nausea in the chair, caused by the rubber dam, etc., phenol-sodique is recommended. Dilute one teaspoonful in about half a glass of water and have the patient take a swallow; repeat in ten or fifteen minutes, if need be.—Brief.

To Cement Arsenic In Cavity Without Pressure.—Mix the cement rather thin and place a small drop on a small bit of paper and carry the paper to the cavity with the pliers. Press to place with a burnisher. The paper facilitates adjustment to place and prevents cement adhering to instrument.—C. B. Warner, Avon, Ill., Tri-State Dental Quarterly.

Dentist Sues Russian Prince.—Prince Nicholas W. Engalitcheff, imperial Russian vice-consul in Chicago, has been made the defendant in a suit for $288 brought by Dr. Lee K. Stewart, a dentist, 103 State street, who claims the consul has refused to pay that amount for work done for his wife in 1905. The jury returned a verdict for the plaintiff.—Chicago Herald.

What can be done by treatment consists of laying patient on his back and give inhalations of ammonia or of amyl nitrite. But my opinion is that if dentists would study cocain, its effects and after-effects, and would live up to it, there would be no danger from its use directly or in preparations put out by the several houses.—Dental Brief.

After a careful study of this annoying class of dentures, I have come to a positive conclusion that weight beyond that which is absolutely necessary in the construction of any given class of denture is a detriment instead of an advantage, as far as its utility is concerned.—Dr. W. M. Bartlett, Summary.

The porcelain-like nature of Ascher's Enamel demands bulk for strength, and this means that margins must not be beveled as for gold fillings. Because the enamel is packed in as a filling, it is unnecessary to extend margins as required in inlay work, and being very adhesive it supports weakened walls rather than strains them by its insertion, hence there is a saving of tooth structure in cavity preparation.—Dr. C. M. Baldwin, Amer. D. Journal.

Carborundum Powder.—When carborundum wheels, disks, etc., become clogged with amalagam or worn too smooth to cut, they can be speedily restored to usefulness by dipping in water or glycerine and then in carbo-powder, using them, repeating the process same as using copper-carbo disks. Disks of all kinds will cut much faster with the powder than new ones will without it. Try it.—J. J. Rapp, Summary.

For Perspiration of the Feet.—Wash the feet with a weak formaldehyde solution (1 part liquor formaldehyde to 9 parts of water); then dust with a powder of the following composition:

Rx. Salicylic acid, 2 drams;
Burnt alum, 2 ozs.
Tannic acid, 2 drams.
Purified talc. 4 ozs.
Oil of bergamat, 10 drops.

Rub well into the feet every night and shake into the stockings every morning. Change stockings every day.—Critic and Guide.

To Whom Dental Licenses are Issued in Japan.—Ambassador Luke E. Wright, of Tokyo, transmits translations of recent ordinances relating to licenses for the practice of medicine and dentistry in Japan. The Government will grant licenses for their practice to graduates of foreign medical and dental colleges, or those who have practiced such professions in foreign countries, who may satisfy the Japanese Government requirements.—Consulor and Trade Reports.

Acute Inflammation of the Peridental Membrane.—Having assurance that the apex of the root is open, place in the pulp chamber a piece of solidified formaldehyde the size of a pinhead and seal in; relief will come, if not immediately, within a few hours, all causes of congestion being completely killed.—F. B. Lawrence, Western Dental Journal.

To Stop Pain Caused by Formalin and Creosote.—Occasionally during treatment a small amount of the formalin-creosote preparations commonly used for treatment will by accident come in contact with the mucous membrane of the mouth and therefore cause severe pain. A pledget of cotton saturated with a 3% solution of eucaine and applied to the surface affected will give immediate relief.—ROY C. ROWLEY, D. D. S., Brief.

DRAINAGE OF ABSCESSES.—In all cases of abscesses in the mucous tissues of the mouth I insist on the use of 95 per cent carbolic acid on the tent. A very small amount retained in the gauze or cotton is sufficient to cauterize the lips of the incision, making the opening freer, promoting the egress of pus and preventing the ingress of new infective material.—G. V. BLACK, Northwestern Dental Journal.

SETTING A SHELL CROWN.—To get the closest adaptation drill a hole through the thickest part of the cusp, tap it and fit a piece of threaded gold wire. In setting the crown remove the wire and allow the surplus of cement to escape through the hole; when the cement is nearly hard clear it away from the hole and screw in the gold wire. When the cement is thoroughly hard cut the wire flush and polish.—R. M. SANGER, Items of Interest.

Platinum, which bounded up in price several times last year, and kept on the upward movement the first three months of this year, began two weeks ago to turn the other way, and since then has fallen $4 an ounce, going down from $38, the top mark, to $34. Refiners say that there is a likelihood of a further decline.—Exchange.

STERILIZING THE FORCEPS.—The forceps, including the handles, are boiled, in a solution of common washing soda, about a quart of water to a piece of soda the size of a walnut; and there is no rust. A mixture of vaseline and carbolic acid is rubbed on the joints while they are hot, which keeps them as good as new.—F. E. GARNER, British Dental Journal.

REPAIRING AN AMALAGAM FILLING.—To repair an amalagam filling, dry it thoroughly, freshen the surface and use a little soft amalagam at first, build it as you wish being careful to see that the occlusion does not displace it before it sets. If you doubt this just freshen the surfaces of two old fillings and after allowing it to set try and break the joint.—Dr. W. A. Robertson, Review.

Impacted Third Molars.—Paint the inflamed tissues with the following combination:

Rx. Zinc iodid....................................dr. iii
Distilled water..............................oz. i
Tinct. iodin.....................................dr. v
Glycerin to make...........................oz. iv

—Prinz, Dental Digest.

Vulcanizing Rubber.—I believe that the majority of rubber dentures are vulcanized at too high a temperature and in too short a time. A denture should be vulcanized at 280 or 290 degrees—depending upon the rubber—for three hours after the vulcanizer has reached that point. In that time a thick lower denture can be vulcanized solid; you can saw right through the mass and polish the cut surface, but you cannot do that if vulcanized at 320 degrees for 55 minutes approximately.—Dr. Gritman, Dental Cosmos.

Backing Porcelain-Faced Crowns.—By the following method a perfect backing can be secured in only one-third the time that it takes to bend up and burnish. Having ground the tooth at the back to the desired shape, dust it with French chalk and press it firmly with Mellotte's moldine. Draw away the tooth and insert in the pin holes pieces of steel, iron or copper wire about the same gauge as the platinum tooth pins; pour the fusible metal, draw away the die; dust with French chalk and take counter die. You can then strike up the backing without fear of breaking the tooth.—W. B. R., Elliott's Quarterly.

Bubbles in Porcelain.—The reason we have so many bubbles in porcelain bridge work is on account of using too large quantities of porcelain at one time over a rigid framework. The first bake should never cover the pins, or even touch them; if it does, on shrinking it leaves an air space beneath, which later means a bubble. Apply and bake several times, until the framework is covered, bringing to a low glaze each time, and you will have porcelain free from bubbles.—A. W. Starbuck, Western Dental Journal.

Silver Nitrate: Precaution.—After cleansing the teeth, and in all infectious conditions of the gums and oral cavity, I use very frequently a 10 per cent. silver nitrate solution, applied on a small swab. To prevent and neutralize the escharotic effects I first apply tincture of iodin liberally over the gums and about the teeth (but not in the cavities, as the teeth would discolor). I also follow the silver nitrate with more iodin, which prevents any free silver nitrate remaining. The surplus of tincture of iodin is quickly washed out and is only irritating.—Otto Hollinger, Dental Review.

The Third Molar.—The third molar can be best saved by the use of a combination cement and amalgam filling, working the amalgam into the soft cement, giving special care to the edges. The cement affords adhesion, avoids the use of deep undercuts and lessens the susceptibility to thermal changes. Over the deep-seated portions be sure to place a thin gutta-percha layer, as the oxyphosphate is liable to be irritating. I put in many of these fillings, not to avoid expense, but because I know it is best adapted to the case.—John A. Schmidt, Dental Cosmos.

Controlling Hypersensitive Palate When Taking Impressions.—In a case where no one had been able to get impressions, the throat and palate being so sensitive, impressions were taken with no unpleasant symtoms whatever after the following treatment: Three powders of chloretone, each containing five grains, were given to the patient with instructions to take one upon getting up in the morning; another two hours later, eating a very light breakfast; the third after breakfast, before reporting at the office. Two grains were then given at the time of taking the impressions.—A. E. Franklin, Dental Register.

Office Building for Physicians and Dentists.—Prominent physicians and dentists of Indianapolis have completed arrangements for the incorporation of a stock company to erect a handsome office building at a cost of $100,000. It will be owned, operated and occupied by members of the stock company. The building will be five stories high, with exterior of white terra cotta. Care will be taken to make it strictly fireproof and thoroughly modern. The first floor will be occupied by a drug store, a surgical instrument salesroom and clubrooms for the physicians and dentists having offices in the building. In the basement will be a Turkish bath establishment, a barber shop and a medical gymnasium.—Indianapolis News.

Dentistry in the Harem.—Dr. S. M. Bostwick has left for Morocco under a royal commission from his Highness, Muley Abdul Aziz, Sultan, to attend the dental work among the women of the Imperial harem. He left Gibralter for Tangier, and later was met by a caravan of seven men, seven camels and seven mules for the start over the desert. The desert trip takes seven days. Dr. Bostwick will remain as the guest of the Sultan for two months, during which time he will fill the royal molars, as well as those of the harem.—Telegraphic News.

Just Appreciation of Services Rendered.—The Editor just received the enclosed letter, which speaks for itself: Dear Doctor: It gives me great pleasure to endorse you as an adept in the science of relieving pain and much swelling in the mouth and pocketbook. When I just started your treatments my face was swollen and I could not close my pocketbook. After twelve or fourteen months of said treatment, both troubles have been removed. Yours for another treatment.—W. H. B., Dentists Mag.

If dentists were thrown together more they would learn to know each other, and instead of guessing from long range that the other fellow was stuck up or a rascal he would either know that he was or was not, and we believe as a rule the object of our long range guess would prove to be a very good and honest fellow. Many honest differences disappear when men are brought face to face frequently. We intend this as an argument in favor of local societies.—Texas Dental Jour.

There are three conditions in which we find the dental pulp as it is presented to us for treatment; first, pulps which are normal—that is, whose blood and nerve supply are operative; second, pulps whose circulatory system is active, but whose nervous system is deficient; third, pulps whose nervous system is operative but which are devoid of blood. The first class should be devitalized by use of the hypodermic needle and cocain solution, the second class by pressure anæsthesia and in the third class, arsenic should be used.—Dr. W. A. McHenry, Brief.

Cement Fillings: Contact Point.—Clinical evidence indicates that a cement filling in any occluso-proximal cavity should have a much larger contact surface presented to the adjoining tooth than merely a buckling point of contact. The cement should be packed firmly directly against the proximal surface of the adjoining tooth, and in finishing an interdental space created at the cervical third only. After a few hours, floss silk can be carried through to the cervical space and withdrawn laterally, and food will not be forced against the gum septa for a series of years if the cement has been mixed properly stiff.—W. V. B. Ames, Dental Summary.

Strength of Porcelain.—When porcelain is closely imbedded in the cement it is almost impossible to break the edge without first wearing away the cement. I believe it has more strength in that way than any other substance. You can take a crystal from your watch and break it in your fingers, but when it is imbedded in cement as an inlay is, you can stand on it with your full weight, and not break it.—Dr. C. F. Rodolph, Review.

Do Not Varnish Inlays After Setting.—The varnishing of an inlay after setting, for the purpose of keeping the moisture from the cement, by almost all inlay workers has proven incorrect. The cements that are used today in setting inlays are what are called hydraulic cements. We have better success with those cements, for after a proper crystallization has taken place the moisture is immediately allowed to flow over.—W. H. Dudworth, Review.

Small pulp canals can be opened by using a five or ten per cent. solution of hydrochloric acid, working with broaches and finally neutralizing with bicarbonate of soda. The canal being in a sterile condition should be filled with a cement made of creosota, formalin and thymol, or iodoform, oil of cassia and creosote. The dressing should be sealed in the canal with gutta-percha to exclude the saliva and to keep in the vapors which are formed in either antiseptic dressing, as they are of a volatile nature.—Dr. F. P. Rutherford, Brief.

Sensitive Dentin.—The use of pressure with obtundents marks a new era in the treatment of sensitive dentin. Carbolic acid, chloride of zinc or trichloracetic acid, in full strength solutions, applied to the desiccated and protected cavity, on a pledget of cotton wool, is covered over with a thick layer of unvulcanized rubber, and pressure applied by means of a flat ended instrument for a minute or two, when an area of insensitive dentin will ordinarily be found to have been secured. A second application may be required at greater depth of excavation.—Wm. Simms, The Dental Record.

Death from Alveolar Abscess.—When not caused directly from extraordinarily high temperature, death from alveolar abscess is due to septicemia—the direct result of poisoning of the general system by products absorbed from the area of suppuration. Deaths from this cause should be reported in our dental journals. It is a fact that deaths do occur from this cause, and there is a certainty that they will occur in the future. This should be recognized by both the dentists and the people whom they serve.—G. V. Black, Northwestern Dental Journal.

Ethyl Chlorid.—There is a special field, I believe, for ethyl chlorid in cases of short operations upon persons who are not good subjects for nitrous oxid. As far as my own experience goes, it is in the cases of alcoholics of the muscular, high-colored, thick-necked men that ethyl chlorid is particularly valuable. In such cases, I believe, if the dose is properly regulated, it can be safely given in the sitting position.—Dr. J. Blumfield, Dental Brief.

Expansion of Plaster of Paris Casts and its Compensation by the Contraction of Zinc Dies.—The expansion of plaster can be readily perceived by anyone who is called upon to employ this material in the laboratory. I will give you a little of my personal experience. Out of seven plaster impressions taken of five different mouths, dies made of low-fusing metal resulted in five misfits. Of five plaster impressions of the same five mouths—taken as most of us take these plaster impressions—zinc dies made from those plaster casts resulted in five fits; that is to say, the patients could wear the plates constructed upon these dies. We all know that zinc shrinks considerably, and the shrinkage of the zinc just about compensated for the expansion of the plaster.—Dental Register.

Ideal Separating Fluid.—Pearline makes an ideal separation material, dissolved and colored with red ink or a bit of indelible pencil. A dash of the powder into the boiling water for removing the wax from an investment acts like magic; or for cleansing the hands first lather them with a good toilet soap and on to that apply some of the powder, work it up thoroughly, getting it into all crevices, then apply the brush or sponge with water and the hands are clean before you know it; dry them and apply not more than four or five drops of two parts glycerin and one part Pond's Extract perfumed and your hands are ready to work for anybody.—Cosmos.

Manipulating Moldable Porcelain.—I believe that the model method is preferable for adjusting the material.

There are many accessible cavities that can be kept dry for a reasonable length of time, and it can be done much more rapidly by molding directly to the cavity. In the class of cases where it is indicated, however, I think it is desirable to make a model. You merely prepare the cavity, take an impression and dismiss the patient. If your model is not perfect, the final fitting may be advantageously made in the mouth. The fitting is done directly to the cavity, and the questionable feature of the accuracy of the model is eliminated.—F. E. Roach, Chicago.

Cocain and Adrenalin.—Since the introduction of the cocaine-adrenalin combination for the purpose of mitigating pain in operative dentistry, local anesthesia has achieved results which, considering its safety and reliability, cannot be obtained with cocaine or any of its substitutes. From our investigations into the nature of the above combination, we are convinced that it is the safest and most effective means of producing local anesthesia upon a rational basis so far known.—Stomatalogist.

(Some practitioners dissent from this assertion. Editor.)

New Form of Local Anesthesia.—Permit me to call attention to a very interesting feature connected with the tablet used in the production of the Abbot-Lamphear form of anesthesia. The formula of this is:

| | |
|---|---|
| Chemically pure hyoscine hydrobromide | gr. 1-100 |
| Chemically pure morphine hydrobromide | gr. 1-4 |
| Cactin (from cactus grandflorus) | gr. 1-67 |

It is essential that the hyoscine be free from apoatropine and atroscine—because it has been shown that commercial "scopolamine" (chemically identical with hyoscine) is dangerous as well as ineffective when its optical rotation is less than minus 10 degrees; that is, it is,not strongly laevorotatory when there is present much apoatropine; so a preparation from a reliable manufacturer only must be employed. The nearer the hyoscine is to—20 degrees in its optical rotation the better and safer the drug.—Critic and Guide.

BURNISHING THE MATRIX.—One feature about the burnishing of the matrix may be new to some of you. We all know it is difficult to force platinum or even pure gold thoroughly into a deep cavity. Now, it is a very simple matter, and only takes a few seconds to prepare a piece of orange wood or pine, approximating the shape of the cavity, start your matrix over that and get it drawn down so it will fit down into the deepest part of the cavity before you place it into the cavity at all; then by means of pledgets of cotton, force it to the bottom of the cavity and start at that point to do your burnishing,—at the deepest part of the cavity, and work toward the walls, completing the burnishing at the enamel margins.—A. W. STARBUCK, Wes. Journal.

"ANTE-MORTEM" APPRECIATICN.—Some of the most popular and appropriate gatherings of today, as far as dentists are concerned, are the number of banquets given in honor of men, who have by their personal and professional life demonstrated their worthiness. Every state has men who are entitled to honor and the time to honor them is while they are here to participate with you. The acceptance of loving cups, honors and kind words is apt to be more agreeable to all interested, if the recipient is around in health and spirits, rather than when the only spirit is that contained in the embalming fluid. The complimentary dinners given to Drs. Wm. Jarvie, R. Ottolengui, G. V. Black and J. D. Patterson, indicate that the custom is spreading, and let us hope in every community the worthy man, or men, may be likewise honored, and made to feel the world appreciates their true value.—J. P. R., Western Dental Journal.

HOW TO TAKE A BITE THAT MUST BE CORRECT.—Nervous people are unable to close their teeth twice alike. If you wish to articulate a gold crown only, it is hard to have the patient articulate correctly. In full dentures it is almost impossible to get the right bite, as most patients will throw their lower jaw forward.

My way of taking the bite is as follows: Put your patient in a comfortable position, just as when you wish to converse with him. Prepare a trial plate in the ordinary manner with a piece of warm wax upon it. At the heel of the plate put a clutch made of wax. Tell your patient to put the tip of the tongue within the clutch and close the lips. The patient is unable to throw the lower jaw forward or sideways. The reason I ask him to close the lips is because when a patient needs a full upper and lower set of artificial teeth, he has no teeth to close, and by closing the lips he is not so apt to protrude the mandible in case he lifts the tongue out of the clutch.—Richard Kessel, Items of Interest.

Taking an Impression.—Building up a ridge of wax at the posterior edge of the floor of the tray, so that when the tray is in positon in the mouth the wax shall come in contact with the soft palate, just posterior to the hard palate, or a trifle back of the place where you wish to establish the plate line, so that when you have the tray in the mouth, place the wax against the tissue and swing the tray up in position in front, as if it were hanging on a hinge at the back.

The wax is very important, answering two purposes; first, preventing the plaster from running over the heel of the tray into the throat, and forcing it to run over on the sides and in front where you wish it to go. Also the pressure of the wax slightly compresses or raises the soft tissues of the palate and holds it rigid, so that if the patient should swallow or contract the palatal muscles, the plaster will not be pulled down, and also giving the plate made on a model from an impression taken in this way a tighter fit across the heel.—Dr. J. A. Bullard, Review.

Testing a Furnace.—Every user of an electric furnace should provide for testing his muffle in case of failure to operate. This is easily done by taking a few feet of the ordinary electric light wire connected with a plug at one end and a small copper or brass point on each of the other ends. One of these wires should be cut and a lamp put in. The lamp will light up when points are brought into contact with the two terminal wires in the muffle if the wire is not burned out or broken. Some such arrangement will also prove to be a very great help in locating a break in case of repair. Where the wire has burned out there will usually appear a spot, but a break will not show and a tester is indispensable. In the majority of cases the burn will occur within three or four inches of the negative end of the wire, due to the excessive heating of the wire at that point.—DR. F. E. ROACH, Dental Review.

